# High Performance and Recyclable Polypropylene/Styrene–Ethylene–Butylene–Styrene Blends for Next Generation Cable Insulation with Enhanced Breakdown Strength Through Controlling Crystallinity

**DOI:** 10.3390/polym17101361

**Published:** 2025-05-16

**Authors:** Chae Yun Nam, Jun Hyung Lee, Min Ah Kim, Ho Gyu Yoon

**Affiliations:** 1 Department of Materials Science and Engineering, Korea University, 145, Anam-ro, Seongbuk-gu, Seoul 02841, Republic of Korea; namchyoon@korea.ac.kr (C.Y.N.);; 2 Iljin Electric Co., Ltd., 905-17 Mannyeon-ro, Hwaseong-si 18365, Republic of Korea

**Keywords:** polypropylene, styrene–ethylene–butylene–styrene, power cable, insulation material

## Abstract

Reducing the environmental impact is a key reason for developing recyclable insulation materials for high-voltage industries. In this study, polypropylene (PP) blends were prepared via melt mixing with styrene–ethylene–butylene–styrene (SEBS), a thermoplastic elastomer, to improve breakdown strengths at various cooling speeds. A systematic investigation was conducted to evaluate the influence of crystal size, degree of crystallinity, and nucleation growth rate on the breakdown strength. Crystallization behavior was analyzed using isothermal and non-isothermal methods based on the Avrami model. Increasing SEBS content reduced crystallinity, with the lowest nucleation growth rate observed at 35% SEBS. Breakdown strength correlated with crystallization behavior and was further validated by Weibull distribution method. Notably, PP/SEBS blends containing 35% SEBS exhibited the highest breakdown strength of 66.4 kV/mm at a cooling speed of 10 °C/mm. This improvement reflected a reduction in the degree of crystallinity from 36.0% to 22.9% and the lowest growth rate constant (k) at 35% SEBS. Furthermore, the predicted lifetime of PP/SEBS blend containing 35% SEBS, calculated using the oxidation induction time and the Arrhenius equation, was 42 years. These findings demonstrate that SEBS content and cooling rate effectively modulate crystallization and breakdown strength, enabling recyclable PP/SEBS with XLPE-comparable performance for sustainable high-voltage insulation.

## 1. Introduction

The high-voltage industry must continuously develop and modernize electrical grid systems to ensure stable, cost-effective, and environment-friendly power solutions. A key objective is the transmission of energy across both sea and land using extruded polymer-based cables. To enhance the operating voltage and electrical performance of these cables, next-generation cable insulation materials must be developed [1]. Modern extruded high-voltage cables primarily use cross-linked polyethylene (XLPE) for insulation owing to its excellent dielectric, thermal, and mechanical properties, making it an ideal choice for cable insulation applications [2,3]. However, the crosslinking process converts XLPE into a thermosetting material, rendering it nonrecyclable at the end of its service life. With increasing environmental concerns, the need for new eco-friendly insulation materials for cables has become increasingly urgent [4]. Numerous studies have explored alternatives to XLPE. Ethylene vinyl acetate (EVA) copolymer blended with high-density polyethylene (HDPE) has been investigated as a potential solution, with a focus on the effects of varying the EVA content in EVA/HDPE blends [5,6]. However, ethylene-based materials have relatively low working temperatures, posing challenges for high-voltage insulation applications [7]. To address this, studies have focused on polypropylene (PP). PP is a promising cable insulation material owing to its high melting temperature and excellent mechanical properties. Crucially, as a thermoplastic, PP does not require crosslinking and is fully recyclable. Studies on syndiotactic PP have demonstrated that it meets the necessary AC breakdown strength as well as the mechanical and electrical properties required for cable insulation. However, its higher cost compared to isotactic PP (iPP) and polyethylene (PE) limits its widespread use [8,9]. Additionally, the inherently high stiffness and brittleness of PP present further challenges, necessitating modifications to enhance its suitability. Blends of iPP with polyolefin elastomer (POE), an ethylene–octene copolymer, have been developed to address these limitations, with varying POE contents achieving properties comparable to those of XLPE. However, these blends exhibit space charge accumulation and restrict their practical use [10]. Research has also explored PP blends with various thermoplastic elastomers (TPE), including ethylene–propylene–diene monomer (EPDM), ethylene–propylene random copolymer (EPR), and styrene–ethylene–butylene–styrene (SEBS) block copolymer [11,12]. TPEs are a class of copolymers or polymer blends that combine thermoplastic and elastomeric properties, offering advantages of both rubber and plastic materials. Their moderate elongation and ability to return to their original shape contribute to longer lifespans and superior physical performance compared to other materials. However, polymer materials inevitably degrade when exposed to heat, light, water, and oxygen, leading to a significant deterioration in their electrical and mechanical properties. Therefore, it is important to predict the lifetime of a material. Several methods have been proposed to predict the lifetimes and understand the degradation behavior based on accelerated aging studies [13,14]. Peinado, et al. demonstrated that the hydrogenating olefinic double bonds significantly enhances resistance to oxygen and ozone attacks [15]. Yazdani-Pedram et al. developed PP/EPR blend composites that exhibited an eight-fold increase in impact strength (356 J·m^−1^) and a three-fold increase in elongation at break (789%) with 30 wt.% EPR content, compared to pure PP [16].

Breakdown strength is another important factor for insulating materials. When PP is blended with other materials to enhance its mechanical properties for cable manufacturing, its breakdown strength is typically reduced to less than 60% of that of XLPE [17]. This reduction is attributed to the high degree of crystallinity (DOC) of PP. Dielectric breakdown has been reported to occur at spherulite boundaries within insulation materials and is significantly influenced by crystallinity and crystal size [18,19]. As the crystallization of polymer blends is affected by the nature of the blended materials, crystallization kinetics studies are crucial for predicting the development of crystalline structures within the blends. A study on PP/TPE blends demonstrated that rubber components influence the crystallization process, subsequently affecting the nucleation mechanism, and thereby the nucleation density and growth of spherulites. Consequently, the size, morphology, and DOC of the spherulites strongly affect the electrical and mechanical properties of the blends [20]. Controlling the DOC and crystal size remains a significant challenge for using PP as a cable insulation material.

In this study, we propose a novel PP/SEBS blend as an eco-friendly cable insulation material. SEBS, a type of TPE, has been used to reduce PP crystallization and improve its mechanical properties. The effects of crystal size, DOC, nucleation growth rate, and crystalline properties on the breakdown strength of the PP/SEBS blends were examined at different cooling speeds. Both isothermal and non-isothermal crystallization processes were analyzed using the Avrami equation and a non-isothermal crystallization kinetic equation. In addition, the breakdown strength as a function of cooling speeds was analyzed using Weibull distribution analysis to determine the probability of occurrence and analyze the breakdown strength reliability. Finally, the lifetime of the PP/SEBS blend was predicted based on oxidation induction time (OIT) and the Arrhenius equation.

## 2. Experiment

### 2.1. Materials and Sample Preparation

Commercially available PP (EP300C) was purchased from Polymirae Co. Ltd., Seoul, Republic of Korea. with a melt flow index of 0.5 g/10 min, density of 0.9 g/cm^3^, and tensile strength of 32 MPa. SEBS (G1645M) was purchased from Kraton Co. Ltd. (The Woodlands, TX, USA), with a polystyrene content of 11.5–13.5%, melt flow index of 2.5–4.5 g/10 min, and tensile strength of 10.3 MPa. The antioxidant (SONGNOX 21 B), which is a blend of phenolic and phosphite types in a ratio of 1:2, was purchased from Songwon Co., Ltd., Ulsan, Republic of Korea. PP/SEBS blends were prepared by kneading at 200 °C for 40 min at 200 rpm using a kneader mixer. Samples with a diameter of 40 mm and thicknesses of approximately 0.5 mm were obtained using compression molding at 200 °C for measuring electrical properties.

### 2.2. Characteristics

The mechanical properties were measured in accordance with ASTM D 638 using a universal testing machine (UTM; UT-100E, MTDI Inc., Yongin City, Republic of Korea) operating at a fixed speed of 50 mm/min at room temperature. The permittivity and volume resistivity were evaluated using a dielectric analyzer (DEA, GmbH CONCEPT40, Novocontrol, Hundsangen, Germany) in the frequency range of 10^−1^ to 10^6^ Hz at room temperature. To minimize contact resistance with the electrodes, the specimens were coated with silver paste. To observe the dispersion of SEBS in the PP matrix, samples were fractured using liquid nitrogen, followed by immersion in toluene at 25 °C for 20 min to remove SEBS. The etched cross-sections were then sputter-coated with platinum and observed using a field-emission scanning electron microscope (FE-SEM, JEOL, JSM-7000, Fukuoka, Japan) with an accelerating potential of 15.0 kV. The crystallinity of the PP/SEBS blends was evaluated using differential scanning calorimetry (DSC, Q60, TA Instruments, New Castle, DE, USA). Samples were heated from 30 °C to 250 °C at 10 °C/min, followed by cooling down to 30 °C at various speeds, and finally reheated to 250 °C at the same scanning rate. For isothermal crystallization, samples were heated to 240 °C at a rate of 10 °C/min and then cooled to 110, 115, 120, 125 °C. AC breakdown tests were conducted using an AC dielectric strength tester with a voltage increase rate of 1 kV/s. Breakdown strength ($E_{B}$) was calculated using the equation $E_{B}=V_{B}/d$, where $V_{B}$ represents the breakdown voltage and d represents the sample thickness at breakdown [21]. Specimens were positioned between two copper ball electrodes with diameters of 30 mm. To prevent surface discharge and flashover before breakdown, the electrodes and specimens were fully immersed in silicone oil during voltage application.

## 3. Results and Discussion

### 3.1. Mechanical and Electrical Properties

To replace XLPE, the current cable insulation material was replaced with an eco-friendly alternative, PP. From an environmental perspective, PP offers notable advantages over XLPE. PP can be easily melted and reprocessed, which allows it to be fully recycled at the end of its service life. In contrast, XLPE cannot be reprocessed once it is manufactured, making it difficult to recycle through conventional methods. As a result, XLPE waste is typically managed through incineration of landfilling, both of which result in environmental concerns. These differences in end-of-like treatment highlight the greater sustainability of PP in cable insulation applications. However, pure PP presents challenges owing to its low elongation, which necessitates the incorporation of SEBS.

**Figure 1:**
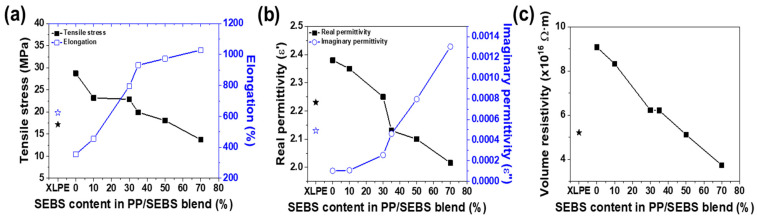
(**a**) Tensile properties, (**b**) real and imaginary permittivity, and (**c**) volume resistivity of PP/SEBS blends as a function of SEBS content. In (**a**), black and blue stars indicate tensile stress and elongation at break, respectively. In (**b**), black and blue lines represent real and imaginary permittivity, respectively.

The mechanical and electrical properties were evaluated based on the SEBS content. As depicted in Figure 1a, pure PP exhibits high strength but low elongation (Figure 1a). With increasing SEBS content in the PP/SEBS blend, the strength decreased, whereas the elongation tended to increase. At 35% SEBS content, the tensile strength of PP decreased from 28.8 to 19.9 MPa, whereas elongation at break increased by 262%.

The elastomeric SEBS acts as a stress concentrator during tensile testing, potentially leading to yielding and crazing in the PP matrix. However, the elastomeric phase can absorb large amounts of energy, mitigating highly strained processes. Permittivity and volume resistivity are key factors for insulating materials, as shown in Figure 1b,c, respectively. AC conductivities of the composite materials were determined using the following formula [22]:

(1) $$\sigma =\omega \cdot \varepsilon^{\prime \prime }\cdot \varepsilon_{0}$$

where ω is the frequency, *ε*″ is the imaginary permittivity, and *ε*_0_ is the vacuum permittivity.

According to this equation, conductivity is proportional to the imaginary permittivity. As SEBS content increases, imaginary permittivity increases, leading to a corresponding decrease in volume resistivity, as depicted in Figure 1b,c. For PP/SEBS blends containing 70% SEBS, the imaginary permittivity and volume resistivity were measured as 0.0013 and 3.74 × 10^16^ Ω∙m, respectively. Figure 1 demonstrates that PP/SEBS blends exhibited properties comparable to XLPE when the SEBS content exceeded 30%.

### 3.2. Morphology

**Figure 2:**
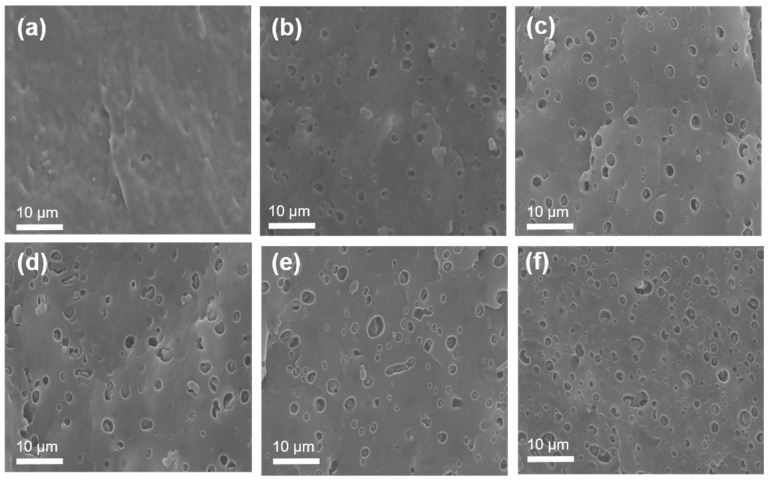
SEM images of PP/SEBS blends with SEBS content: (**a**) 0%, (**b**) 10%, (**c**) 30%, (**d**) 35%, (**e**) 50%, and (**f**) 70%.

The morphology of PP/SEBS blends was examined using cross-sectional analysis to assess SEBS dispersion. Figure 2 shows etched sections of PP and PP/SEBS blends, where round holes indicate the removal of SEBS through etching. In PP/SEBS blends, PP and SEBS are thermodynamically incompatible because of their interfacial interactions. Phase separation occurs owing to differences in viscosity, with lower-viscosity PP forming a continuous matrix and SEBS distributed throughout [23]. Consequently, the distribution of these holes serves as an indicator of SEBS dispersion within the PP matrix, where SEBS forms a sea–island structure with nearly spherical domains approximately 2 μm in diameter. SEBS was uniformly dispersed in PP, but complete compatibility was not achieved. Figure 2f illustrates that at SEBS contents exceeding 35%, some SEBS particles became interconnected, indicating proximity to the brittle–ductile transition point for the blends.

### 3.3. Isothermal and Non-Isothermal Crystallization

**Figure 3:**
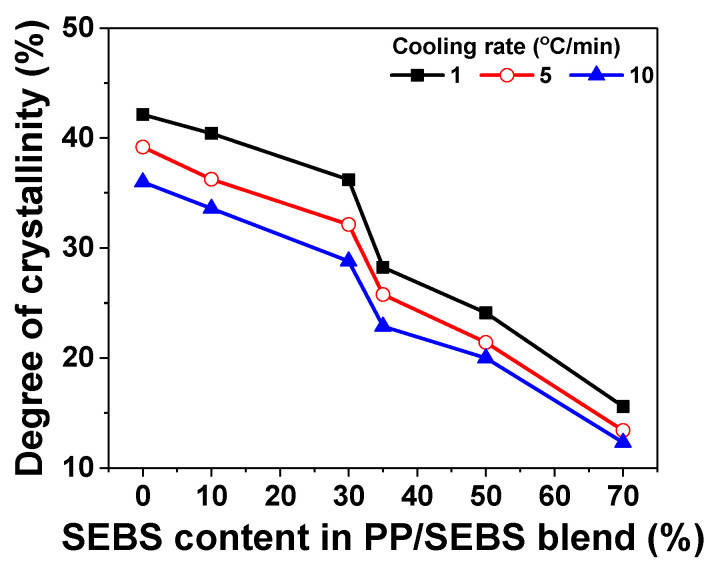
The DOC of PP/SEBS blends as a function of cooling rate.

To evaluate the influence of crystallization on breakdown strength, the crystallization behavior of the PP/SEBS blends was analyzed at various cooling rates. Data such as melting temperature (T_m_), crystallization temperature (T_c_), melting enthalpies (∆*H_m_*), and the degree of crystallinity (DOC, *X_c_*) obtained from the DSC curves of PP/SEBS blends are summarized in Table 1. Figure 3 depicts the variation in the DOC with the SEBS content and cooling rate, following the equation

(2) $$X_{c}\;\left(\%\right)=\frac{\Delta H_{m}}{\emptyset \times \Delta H_{m}^{\infty }}\times 100$$

where $\Delta H_{m}$ is the melting enthalpy of the sample obtained from DSC, $\Delta H_{m}^{\infty }$ is 209 J/g for 100% crystalline PP, and *Ø* is the weight fraction of PP in PP/SEBS blends [24].

Figure 3 illustrates the reduction in the DOC of the PP/SEBS blends with increasing SEBS content, attributed to the rubber-like characteristics of SEBS affecting PP spherulite growth. The entanglement effect of SEBS molecular chains impedes the formation of perfect crystallites by penetrating the lamellar structures, comprising spherulites [25]. Additionally, SEBS disrupts the crystal growth process, resulting in a smaller dimensional distribution. Consequently, DOC in PP/SEBS blends containing 50% SEBS decreased by approximately 55.5%, 54.6%, and 57.2% with increasing cooling rates. These results are consistent with the findings of Uthaipan, et al. [26]. At higher cooling rates, reduced time for spherulite growth leads to lower DOC. The crystal growth rate of PP/SEBS blends was further analyzed to elucidate the rapid reduction in 30% and 35% SEBS content (Figure 3).

The isothermal crystallization kinetics were analyzed by examining the DOC conversion as a function of time at constant temperature. Given the crystallization temperature range of PP/SEBS blends from 107 to 128 °C, isothermal crystallization was studied at 110–125 °C. The variation in crystallinity was correlated with the ratio of heat generated at time *t* to heat generated at infinite time. The relative degree of crystallization, $X_{r}\left(t\right)$, was determined as [27]

(3) $$X_{r}\left(t\right)=X_{c}\left(t\right)\frac{\Delta H_{0}}{\Delta H_{T}}=\frac{\int_{0}^{t}(dH/dt)dt}{\int_{0}^{\infty }(dH/dt)dt}$$

where $\Delta H_{T}$ is the total heat released during crystallization. The crystallization kinetics of the blends were analyzed using the Avrami model, which describes the development of the relative degree of crystallization $X_{r}$ in the isothermal process.

(4) $$X_{r}=1-e^{-kt^{n}}$$

The Avrami exponent (*n*) serves as an indicator of the crystal growth mechanism. The integer value of *n* is indicative of either a homogeneous or heterogeneous nucleation mechanism, and it further defines the type of crystal growth as one-dimensional (rods), two-dimensional (disks), or three-dimensional (cones). The rate constant (*k*) governs the rate of crystalline growth and the nucleation ratio.

These kinetic parameters are determined through the natural logarithmic form of the Avrami model by plotting $\ln[-\ln\left(1-X_{r}\right)]$ against lnt. The Avrami exponent (*n*) corresponds to the slope of the representative curve, while lnk corresponds to its intercept.

(5) $$\ln[-\ln\left(1-X_{r}\right)]=\ln k+n\ln t$$

**Figure 4:**
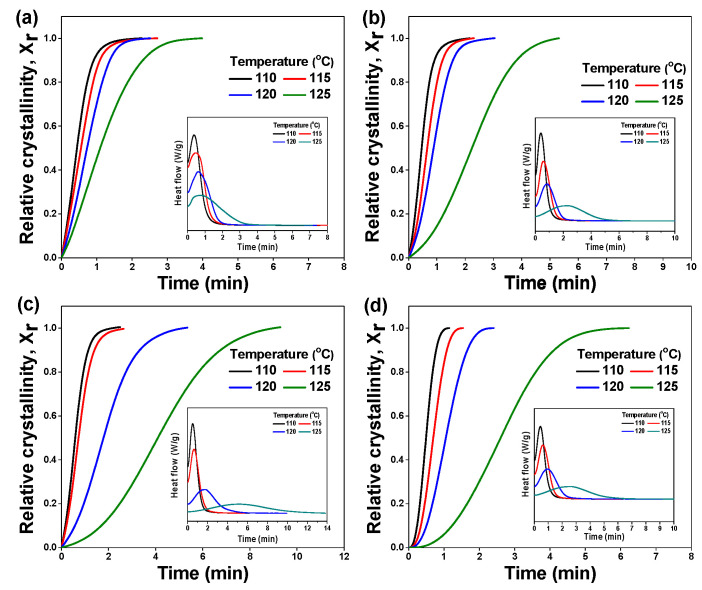
Development of relative crystallinity (*X_r_*) as a function of time of PP/SEBS blends; SEBS content: (**a**) 0%, (**b**) 10%, (**c**) 35%, and (**d**) 50% (inset; isothermal crystallization).

The DSC curve and the relative DOC ($X_{r}$) are plotted against time in Figure 4. From the DSC curve, the crystallization rate is used to determine the time required to achieve 50% crystallization, referred to as the half-time of crystallization ($\tau_{1/2}$), which is calculated from the onset and conclusion of crystallization as shown in Table 2. Higher $\tau_{1/2}$ values indicate slower crystallization rates. Additionally, $\tau_{1/2}$ values exhibit a proportional relationship with temperature in PP/SEBS blends. Notably, the PP/SEBS blend with an SEBS content of 35% demonstrated the highest $\tau_{1/2}$ value, as presented in Table 2. These findings highlight the impact of SEBS on inhibiting PP nucleation and its influence on PP crystallization. The decrease in $\tau_{1/2}$ with increasing SEBS content suggests it may affect spherulite growth [28].

**Figure 5:**
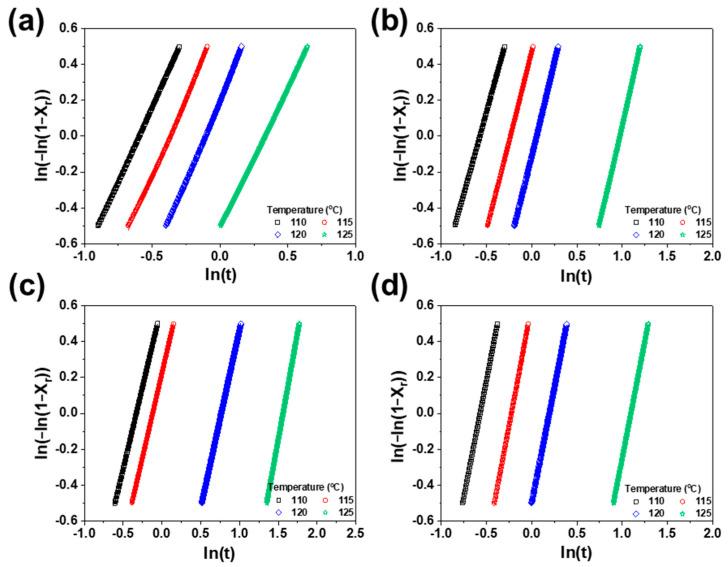
Plot of ln(−ln(1 − *X_r_*)) versus ln*t* for isothermal crystallization. The exponential form of the Avrami model of PP/SEBS blends: SEBS content of (**a**) 0%, (**b**) 10%, (**c**) 35%, and (**d**) 50%.

**Figure 6:**
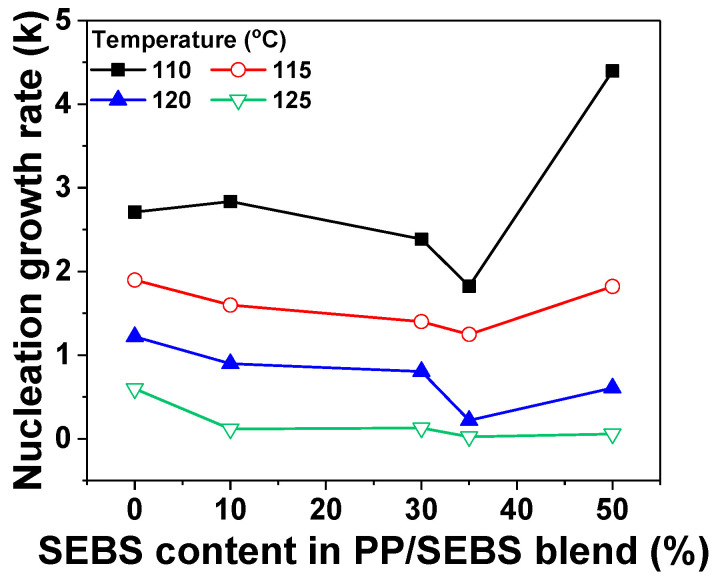
The nucleation growth rate of PP/SEBS blends with varying SEBS content.

The findings derived from the Avrami model analysis are shown in Figure 5. The nucleation growth rate (*k*) of the PP/SEBS blends clarifies the effects of rubber materials on crystal growth rate and nucleation density. The reduction in *k* for the PP/SEBS blends indicates that SEBS inhibited the spherulite growth of PP. The relationship between crystallization temperature, SEBS content, and nucleation growth rate (*k*) (Figure 6) shows that lower crystallization temperatures correspond to higher nucleation growth rates, while an increase in SEBS content results in lower crystallization rates. Abbasi et al. suggested that the crystal growth rate has a nonlinear dependence on rubber content [29]. Notably, at an SEBS content of 35%, the rate constant reached its minimum value, suggesting that a lower nucleation growth rate corresponds to smaller crystal sizes at the same DOC. Another factor, the Avrami exponent (*n*), provides insights into the influence of rubber materials on nucleation and morphology. Typically ranging between 2 and 3, an increase in *n* indicates a transition from predominantly homogeneous to heterogeneous nucleation mechanisms in the PP [30]. The presence of SEBS induced a slight decrease in *n*, suggesting that SEBS affects the overall morphology of the PP crystal structure.

### 3.4. Breakdown Strength

**Figure 7:**
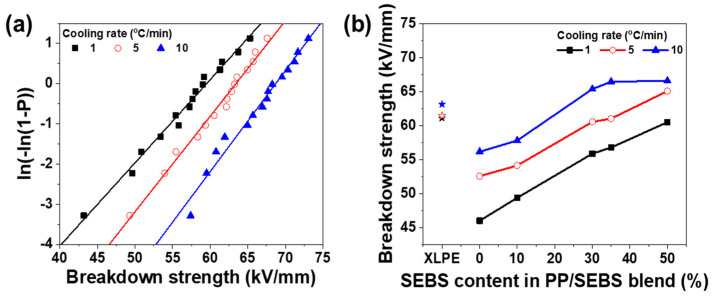
(**a**) Weibull plots of AC breakdown strength of PP/SEBS blends as a function of cooling rate at 35% SEBS in blends and (**b**) breakdown strength of PP/SEBS blends with varying SEBS content.

Breakdown strength measurements were conducted to validate the influence of DOC and nucleation growth rate on the insulation properties. The breakdown–strength distribution data were analyzed using the Weibull probability distribution. The experimental data on the AC breakdown strength were subjected to a Weibull statistical analysis, expressed as follows:

(6) $$P\left(E,\;\alpha ,\;\beta \right)=1-\exp\left[-{\left(\frac{E}{\alpha }\right)}^{\beta }\right]$$

where *P*(*E*, *α*, *β*) represents the breakdown probability, *E* is the measured breakdown strength, *α* is the characteristic breakdown strength at 63.2% cumulative breakdown probability, and *β* is the shape parameter indicating data scatter. According to IEEE Standard 930-2004 [31], a simple method for approximating the cumulative breakdown probability can be described as follows:

(7) $$P_{i}=\frac{i-0.44}{n+0.25}\times 100\;\left(\%\right)$$

where $P_{i}$ is the cumulative probability of *i*th breakdown data point, *i* represents the breakdown strength values arranged in ascending order, *n* is the total number of samples, which is 15 in this study. The breakdown strengths of the specimens are depicted in Figure 7, and the characteristic breakdown strengths and shape parameters are listed in Table 3. Pure PP exhibited a breakdown strength of 45.9 kV/mm at a cooling speed of 1 °C/min, the lowest breakdown strength among pure PP samples tested. Notably, the breakdown strength of PP/SEBS blends containing 35% SEBS peaked at 66.4 kV/mm, marking a significant increase of approximately 118.3% compared to pure PP at a cooling speed of 10 °C/min. The highest breakdown strength observed in the PP/SEBS blends may be ascribed to the presence of aromatic rings in SEBS, which reduce the energy of carriers injected from the electrodes. Consequently, this decreases the probability of molecular chain breakage by high-energy carriers, enhancing breakdown strength [32]. According to Fan et al., breakdown occurs along spherulite boundaries and is influenced by both the DOC and crystal size. Smaller DOC and crystal sizes correspond to higher breakdown strengths [33]. The shape parameter (β) reflects the failure rate, which is linked to data dispersion. A narrower distribution of the breakdown strength curve is indicative of a higher β value, signifying improved data reliability. As detailed in Table 3, the β values for PP/SEBS blends generally exceed 10. The PP/SEBS blend containing 35% SEBS content shows the highest β value, with β increasing alongside cooling speed. These results demonstrate that breakdown strength is highest for PP/SEBS blends with 35% SEBS content. This trend indicates that breakdown strength improves as DOC and crystal size are controlled.

### 3.5. Accelerated Lifetime Test

**Figure 8:**
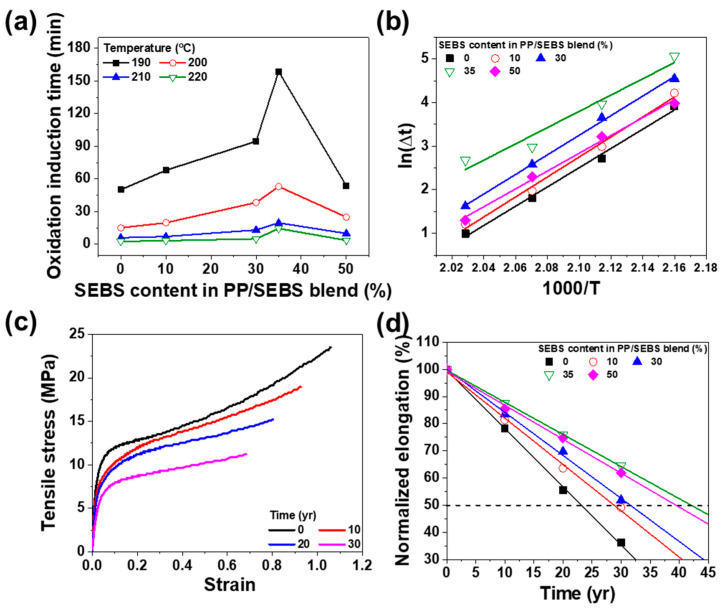
Accelerated life test of PP/SEBS blends as a function of SEBS content. (**a**) OIT, (**b**) Arrhenius plot, (**c**) stress–strain curve of SEBS 35% over aging time, and (**d**) normalized elongation for predicting the expected lifetime.

Polymer materials experience reduced mechanical properties and potential discoloration under prolonged high-temperature environments, a critical concern for cable applications [34]. Analyzing accelerated life prediction is essential for ascertaining the long-term suitability of materials in such environments. Figure 8 illustrates the life expectancy predicted using the Arrhenius model and OIT. The Arrhenius model describes the temperature–lifetime relationship of polymer materials and is given as

(8) $$k=A\cdot e^{-E_{a}/RT}$$

where $E_{a}$ is the Arrhenius activation energy, *R* is the gas constant (8.314 J/mol·K), *T* is the absolute temperature, and *A* is the pre-exponential factor. To practically evaluate the thermal aging behavior and predict the lifetime at different temperatures, the temperature-dependent degradation time or rate data can be conveniently expressed using accelerative shift factors $\alpha_{T}$, which describe the temperature dependency of the degradation process. These shift factors can be normalized for any desired temperature. The equation below facilitates the determination of the activation energy across a specified temperature range [35].

(9) $$a_{T}=exp\left[\frac{E_{a}}{R}\left(\frac{1}{T_{2}}-\frac{1}{T_{1}}\right)\right],\;a_{T}=\frac{t_{1}}{t_{2}}$$

Here, $T_{1}$ is the accelerated degradation temperature, and $T_{2}$ is the real operating temperature. In this study, $T_{1}$ is set to 120 °C as the accelerated degradation temperature at which OIT was measured, and $T_{2}$ is 90 °C, representing the real operating temperature of the cable. By selecting target predicted lifetime $t_{2}$ (i.e., 10, 20, or 30 years), the corresponding degradation time $t_{1}$ at the accelerated degradation temperature can be calculated to estimate the expected lifetime at $T_{2}$.

In accordance with Figure 8a and the equation above, if the Arrhenius behavior holds, plotting the logarithm of the shift factors against the reciprocal of the absolute aging temperature yields a linear relationship, as shown in Figure 8b. Figure 8c illustrates the changes in the stress–strain curve of SEBS 35% with increasing time. As aging progressed, polymer chain degradation led to the breaking of the polymer chains, resulting in a decline in both the tensile strength and strain. The mechanical properties of the aged samples over time are illustrated in Figure 8d, which are used to predict the lifetime. Defining 50% elongation at break as the failure criterion, the predicted lifetime of PP was determined to be 23 years. PP oxidation typically occurs at tertiary carbon centers, causing chain breakage through reactions with oxygen. Zaharescu, et al. proposed that SEBS maintains thermal stability owing to its phenolic structure, which can function as an antioxidant when reacting with diene polymers [36].

**Figure 9:**
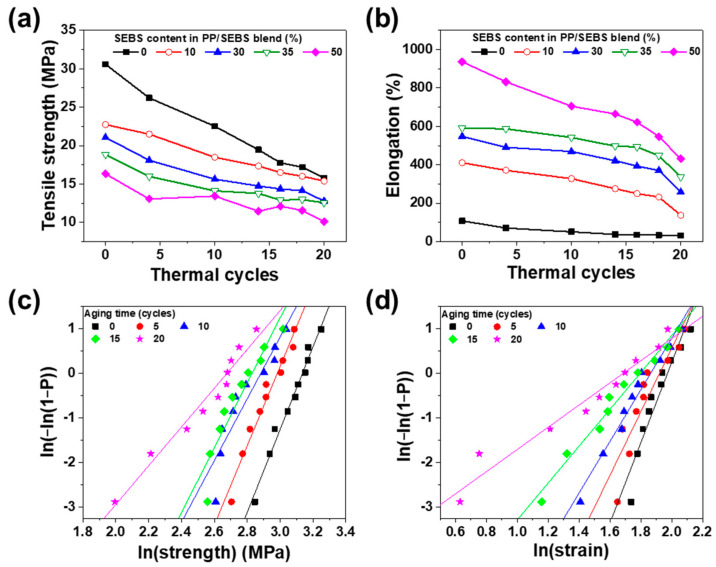
Thermal cycling test under constant thermal stress at 120 °C; (**a**) tensile stress and (**b**) elongation as a function of SEBS content. Weibull plot of (**c**) tensile strength, and (**d**) strain according to aging time for 35% SEBS content in PP/SEBS blend.

To assess the impact of accelerated degradation on the mechanical properties of PP/SEBS blends, changes in tensile strength and elongation were measured under sustained thermal stress at 120 °C over multiple thermal cycles. As shown in Figure 9, after 20 thermal cycles, PP exhibited a 48.4% decrease in tensile strength, whereas the blend with 35% SEBS showed a smaller reduction of 33.4%. Moreover, elongation at break decreased by 70.6% for PP and 43.1% for 35% SEBS under the same conditions. These findings, in agreement with those of Shebani, et al., indicate that TPE enhances the thermal stability of PP [37]. Figure 9c,d illustrate the Weibull distribution of tensile strength and strain for SEBS 35% across thermal cycles. Both tensile strength and strain decreased with increasing thermal cycles, And the Weibull plots revealed a reduction in slope and broader data distribution, indicating diminished stability. This broader distribution is primarily attributed to the scission of the polymer chains during degradation. Notably, the Weibull plot slope serves as an indicator of material stability, with higher slope values signifying greater stability. As shown in Figure 9c,d, the slope decreases with advancing degradation, highlighting reduced stability under thermal cycling. However, the SEBS 35% blend maintained a relatively stable distribution, suggesting that it possesses suitable degradation characteristics for replacing XLPE. Overall, thermal stability improved with increasing SEBS concentration in the PP/SEBS blends, as shown in Figure 8 and Figure 9. Specifically, pure PP demonstrated the shortest lifetime, whereas the longest lifetime of 42 years was achieved with 35% SEBS content.

## 4. Conclusions

This study systematically examined the potential of PP/SEBS blends as recyclable alternatives to XLPE for high-voltage cable insulation. By varying SEBS content and controlling cooling rates, we effectively controlled the crystallization behavior, which led to improvements in breakdown strength.

The mechanical and electrical properties of the PP/SEBS blends were superior to those of pure PP, with a notable enhancement in breakdown strength attributed to factors such as the crystal size, DOC, and nucleation growth rate under different cooling rates. Crystallization behavior, examined through isothermal and non-isothermal processes using the Avrami model, revealed a decline in the DOC and nucleation growth rate with increasing SEBS content. At 35% SEBS, the DOC and nucleation growth rate were 22.9% and 1.69 at 110 °C, respectively. The impact of the crystallinity and nucleation growth rate on the breakdown strength was further analyzed at various cooling rates. The highest breakdown strength of 66.4 kV/mm was achieved with 35% SEBS and a cooling rate of 10 °C/min. Weibull analysis confirmed that the addition of SEBS significantly enhanced breakdown strength by reducing crystallinity and nucleation growth rate. The thermal stability of PP was also improved by SEBS, as demonstrated by the lifetime predictions using the Arrhenius model. Extrapolation indicated that PP/SEBS blends with 35% SEBS could achieve a lifetime of 42 years. A comprehensive analysis indicated that PP/SEBS blends with 35% SEBS exhibited the most favorable mechanical and electrical properties. These blends demonstrated enhanced breakdown strength owing to the optimized DOC, reduced nucleation growth rate, and superior mechanical performance with minimal reductions in tensile stress and increased elongation over aging time.

Importantly, PP/SEBS blends offer significant technological advantages over conventional XLPE. These include melt processability without the need for chemical crosslinking, lower energy consumption, and full recyclability. Such properties simplify production and reduce environmental burden. In terms of insulation formation, the blends exhibit high breakdown strength and long-term reliability, making them practically applicable in high-voltage cable.

In conclusion, PP/SEBS blends with controlled cooling rates exhibited excellent mechanical and electrical properties that surpassed those of conventional XLPE. These findings suggest that the PP/SEBS blends are promising eco-friendly cable insulation materials, offering distinct technological and environmental advantages in terms of processing, recyclability, and insulation properties.

## Figures and Tables

**Table 1:** Summary of DSC data of PP/SEBS blends.

Cooling Rate	Content of SEBS (%)	T_m_ (°C)	T_c_ (°C)	Δ*H_m_* (J/g)	X_c_ (%)
1 °C/min	0	171.30	122.1	88.07	42.14
	10	170.93	120.9	76.03	40.42
	30	170.32	119.5	52.97	36.21
	35	170.05	118.2	38.36	28.24
	50	167.97	116.3	25.19	24.11
5 °C/min	0	170.92	114.6	81.88	39.18
	10	170.02	113.4	68.18	36.25
	30	169.84	111.6	47.03	32.14
	35	169.64	110.4	34.99	25.76
	50	168.72	109.0	22.37	21.41
10 °C/min	0	171.23	116.9	75.25	36.00
	10	170.87	111.8	63.17	33.58
	30	170.13	110.1	42.14	28.81
	35	170.05	108.9	31.07	22.87
	50	169.05	107.7	20.88	19.98

**Table 2:** Kinetic parameters from the analysis of isothermal crystallization of PP/SEBS blends.

Isothermal Temperature	Content of SEBS (%)	τ12	*n*	*k*
110	0	0.42	1.67	2.71
	10	0.44	1.82	2.84
	35	0.56	1.83	1.82
	50	0.49	2.60	4.40
115	0	0.51	1.72	1.90
	10	0.63	1.99	1.60
	35	0.73	1.86	1.25
	50	0.70	2.67	1.82
120	0	0.68	1.80	1.22
	10	0.84	2.07	0.90
	35	1.72	1.98	0.22
	50	1.05	2.57	0.61
125	0	1.05	1.56	0.60
	10	2.17	2.20	0.12
	35	3.79	2.39	0.02
	50	2.62	2.60	0.06

**Table 3:** Kinetic parameters from the analysis of dielectric breakdown strength of PP/SEBS blends.

Cooling Rate	Content of SEBS (%)	*σ* (kV/mm)	*β*
1 °C/min	0	45.99	7.89
	10	49.37	9.10
	30	55.85	11.92
	35	56.76	11.15
	50	60.49	10.89
5 °C/min	0	52.55	9.32
	10	54.11	10.70
	30	60.53	13.42
	35	61.02	13.83
	50	65.06	13.50
10 °C/min	0	56.14	11.46
	10	57.79	12.53
	30	65.36	14.50
	35	66.44	16.38
	50	66.57	14.28

## Data Availability

Data are contained within the article.

## References

[1] Reed C.W. (2017). An Assessment of Material Selection for High Voltage DC Extruded Polymer Cables. IEEE Electr. Insul. Mag..

[2] Meng F., Chen X., Dai C., Zhang M., Paramane A., Zheng L., Tanaka Y. (2021). Effect of Thermal Ageing on Physico-Chemical and Electrical Properties of EHVDC XLPE Cable Insulation. IEEE Trans. Dielectr. Electr. Insul..

[3] Zhu X., Yin Y., Wu J. (2020). Study on Aging Characteristics of XLPE Cable Insulation Based on Quantum Chemical Calculation. IEEE Trans. Dielectr. Electr. Insul..

[4] Zhou Y., Dang B., Wang H., Liu J., Li Q., Hu J., He J. (2018). Polypropylene-based ternary nanocomposites for recyclable high-voltage direct-current cable insulation. Compos. Sci. Technol..

[5] Hosier I.L., Vaughan A.S., Swingler S.G. (2010). An investigation of the potential of ethylene vinyl acetate/polyethylene blends for use in recyclable high voltage cable insulation systems. J. Mater. Sci..

[6] Andrews T., Hampton R.N., Smedberg A., Wald D., Waschk V., Weissenberg W. (2006). The role of Degassing in XLPE Power Cable Manufacture. IEEE Electr. Insul. Mag..

[7] Suh K.S., Kim J.Y., Lee C.R. (1996). Charge Distribution in Polyethylene/Ethylene Vinylacetate Laminates and Blends. IEEE Trans. Dielectr. Electr. Insul..

[8] Li J., Yang K., Wu K., Jing Z., Dong J.Y. (2023). Eco-friendly polypropylene power cable insulation: Present status and perspective. IET Nanodielectrics.

[9] Shirvanimoghaddam K., Balaji K.V., Yadav R., Zabihi O., Ahmadi M., Adetunji P., Naebe M. (2021). Balancing the toughness and strength in polypropylene composites. Compos. Part B Eng..

[10] Zhan Y., Yang X., Yang J., Hou S., Fu M. (2024). Improved electrical properties of organic modified thermoplastic insulation material for direct current cable application. Polymers.

[11] Xu C., Zheng Z., Wu W., Wang Z., Fu L. (2019). Dynamically vulcanized PP/EPDM blends with balanced stiffness and toughness via in-situ compatibilization of MAA and excess ZnO nanoparticles: Preparation, structure and properties. Compos. Part B Eng..

[12] Panaitescu D.M., Vuluga Z., Sanporean C.G., Nicolae C.A., Gabor A.R., Trusca R. (2019). High flow polypropylene/SEBS composites reinforced with differently treated hemp fibers for injection molded parts. Compos. Part B Eng..

[13] Song H.S., Kim H.S., Jung J.H., Lee B.W. (2024). Comparison for Accelerated Degradation of New and Old 6.6 kV AC XLPE Cables. J. Electr. Eng. Technol..

[14] Zhang Z., Assala P.D.S., Wu L. (2018). Residual life assessment of 110 kV XLPE cable. Electr. Power Syst. Res..

[15] Peinado C., Corrales T., Catalina F., Pedron S., Quiteria V.R.S., Parellada M.D., Barrio J.A., Olmos D., Gonzalez-Benito J. (2010). Effects of ozone in surface modification and thermal stability of SEBS block copolymers. Polym. Degrad. Stab..

[16] Yazdani-Pedram M., Quijada R., Lopez-Manchado M.A. (2003). Use of Monomethyl Itaconate Grafted Poly(propylene)(PP) and Ethylene Propylene Rubber (EPR) as Compatibilizers for PP/EPR Blends. Macromol. Mater. Eng..

[17] Hosier I.L., Vaughan A.S., Pye A., Stevens G.C. (2019). High performance polymer blend systems for HVDC applications. IEEE Trans. Dielectr. Electr. Insul..

[18] Wu Y., Li Z., Wang H., Zheng Z., Du B. (2024). Enhanced Dielectric Breakdown Property of Polypropylene Based on Mesoscopic Structure Modulation by Crystal Phase Transformation for High Voltage Power Cable Insulation. ACS Appl. Polym. Mater..

[19] Li Y., Han Y., Pang J., Jin D., Sun Y., Li Z. (2024). Electric Field Assist on Enhancing the Electrical Breakdown Strength of Cross-Linked Polyethylene for Power Cable Insulation. Macromolecules.

[20] Lou C.W., Huang C.L., Pan Y.J., Lin Z.L., Song X.M., Lin J.H. (2016). Crystallization, mechanical, and electromagnetic properties of conductive polypropylene/SEBS composites. J. Polym. Res..

[21] Nazrin A., Kuan T.M., Mansour D.E.A., Farade R.A., Ariffin A.M., Rahman M.S.A., Wahab N.I.B.A. (2024). Innovative approaches for augmenting dielectric properties in cross-linked polyethylene (XLPE): A review. Heliyon.

[22] Das A.K., Chatterjee S., Pradhan A.K., Chatterjee B., Dalai S. (2022). Estimation of moisture content in XLPE cable insulation using electric modulus. IEEE Trans. Dielectr. Electr. Insul..

[23] Banerjee S.S., Burbine S., Shivaprakash N.K., Mead J. (2019). 3D-Printable PP/SEBS Thermoplastic Elastomeric Blends: Preparation and Properties. Polymers.

[24] Li Z., Wu Y., Du B. (2023). Effect of Crystalline Morphology on Electrical Tree Morphology and Growth Characteristics of PP Insulation: From Mesoscopic to Macroscopic. IEEE Trans. Dielectr. Electr. Insul..

[25] Li S., Cheng P., Liu X., Li G., Ma Y. (2023). Fabrication and toughening mechanism of high toughness PP/SEBS/HDPE blends with core-shell particles. J. Polym. Sci..

[26] Uthaipan N., Jarnthong M., Peng Z., Junhasavasdikul B., Nakason C., Thitithammawong A. (2015). Effects of cooling rates on crystallization behavior and melting characteristics of isotactic polypropylene as neat and in the TPVs EPDM/PP and EOC/PP. Polym. Test..

[27] Seo Y., Kim J., Kim K.U., Kim Y.C. (2000). Study of the crystallization behaviors of polypropylene and maleic anhydride grafted polypropylene. Polymer.

[28] Zhang Z., Yu F., Zhang H. (2017). Isothermal and Non-Isothermal Crystallization Studies of Long Chain Branched Polypropylene Containing Poly(ethylene-co-octene) under Quiescent and Shear Conditions. Polymers.

[29] Abbasi A., Abadi A.N., Hemmati F., Mohammadi-Roshandeh J., Farizeh T. (2023). Structure-properties correlations in compatibilized polyamide/thermoplastic elastomer/nanoclay mixtures: Interrelationship among non-isothermal crystallization kinetics, morphology and viscoelastic responses. J. Therm. Anal. Calorim..

[30] Patra P.K., Jaisingh A., Goel V., Kapur G.S., Nebhani L. (2022). Crystallization kinetics of compatibilized blends of polypropylene and polyethylenimine. J. Therm. Anal. Calorim..

[31] (2004). IEEE Guide for the Statistical Analysis of Electrical Insulation Breakdown Data.

[32] Guo Q., Li X., Li W., Yao Z. (2018). The Balanced Insulating Performance and Mechanical Property of PP by Introducing PP-g-PS Graft Copolymer and SEBS Elastomer. Ind. Eng. Chem. Res..

[33] Fan M., Zhou S., Li Z., Du B., Yu F., Yan H. Effect of Crystalline Morphology on DC-Prestressed Breakdown Characteristics of PP-based Cable Insulation. Proceedings of the 3rd IEEE International Conference on Electrical Materials and Power Equipment.

[34] Dai X., Hao J., Liao R., Zheng X., Gao Z., Peng W. (2021). Multi-dimensional Analysis and Correlation Mechanism of Thermal Degradation Characteristics of XLPE Insulation for Extra High Voltage Submarine Cable. IEEE Trans. Dielectr. Electr. Insul..

[35] Shan B., Du C., Cheng J., Wang W., Li C. (2022). Residual life prediction of XLPE distribution cables based on time-temperature superposition principle by non-destructive BIS measuring on site. Polymers.

[36] Zaharescu T. (2025). Insight into the stabilization activity of n-SiO_2_ powder in SEBS phase. J. Therm. Anal. Calorim..

[37] Shebani A., Algoul S., Al-Qish A., Alaeb A., Trish A. (2025). Enhancement of mechanical properties and thermal stability of recycled polyethylene, polypropylene and their blends via incorporation of elastomer. J. Elastomers Plast..

